# Disulfide isomerase ERp57 improves the stability and immunogenicity of H3N2 influenza virus hemagglutinin

**DOI:** 10.1186/s12985-020-01325-x

**Published:** 2020-04-21

**Authors:** Jialing Wu, Yang Wang, Ying Wei, Zhichao Xu, Xin Tan, Zhihui Wu, Jing Zheng, George Dacai Liu, Yongchang Cao, Chunyi Xue

**Affiliations:** 1 State Key Laboratory of Biocontrol, School of Life Science, Sun Yat-sen University, Higher Education Mega Center, Guangzhou, 510006 China; 2 Clinical Research Institute, The First People’s Hospital of Foshan, Foshan, 528000 China; 3 Firstline Biopharmaceuticals Corporation, 12050 167th PL NE, Redmond, WA 98052 USA

**Keywords:** Hemagglutinin, Protein disulfide isomerase, ERp57, Stability, Immunogenicity

## Abstract

**Background:**

Hemagglutinin (HA), as the surface immunogenic protein, is the most important component of influenza viruses. Previous studies showed that the stability of HA was significant for HA’s immunogenicity, and many efforts have been made to stabilize the expressed HA proteins.

**Methods:**

In this study, the protein disulfide isomerases (PDIs) were investigated for the ability to improve the stability of HA protein. Two members of the PDIs family, PDI and ERp57, were over-expressed or down-expressed in 293 T cells. The expression of H3 HA and PDIs were investigated by real-time qPCR, western-blot, immunofluorescence assay, and flow cytometry. The stability of HA was investigated by western-blot under non-reducing condition. Moreover, BALB/c mice were immunized subcutaneously twice with the vaccine that contained HA proteins from the ERp57-overexpressed and conventional 293 T cells respectively to investigate the impact of ERp57 on the immunogenicity of H3N2 HA.

**Results:**

The percentage of the disulfide-bonded HA trimers increased significantly in the PDIs-overexpressed 293 T cells, and ERp57 was more valid to the stability of HA than PDI. The knockdown of ERp57 by small interfering RNA significantly decreased the percentage of the disulfide-bonded HA trimers. HA proteins from ERp57-overexpressed 293 T cells stimulated the mice to generate significantly higher HA-specific IgG against H1N1 and H3N2 viruses than those from the conventional cells. The mice receiving H3 HA from ERp57-overexpressed 293 T cells showed the better resistance against H1N1 viruses and the higher survival rate than the mice receiving H3 HA from the conventional cells.

**Conclusion:**

ERp57 could improve the stability and immunogenicity of H3N2 HA.

## Background

The infection of the influenza virus is still one of the main reasons which cause human illness and death [1–3]. There are four severe outbreaks of influenza, the Spanish influenza in 1918 which was caused by H1N1, the Asian influenza in 1958 caused by H2N2, the Hong Kong influenza in 1968 caused by H3N2 and the Mexico influenza in 2009 caused by H1N1. Today the prevention of influenza virus is still a big challenge because of its high mutability [3, 4]. Vaccine immunization is the most important way to prevent from influenza at present, and vaccines are prepared every year based on the predicted influenza virus strains [2–5]. It will lead to a pandemic if the prediction is inaccurate [4]. So broad-spectrum vaccines with wider protection against influenza viruses have become a hot topic in recent years.

Hemagglutinin (HA), as the homo-trimeric glycoprotein of the influenza virus envelope, contains an extracellular, transmembrane and intracellular region [6]. HA plays an important role in infection by mediating virus entry into the host cell and fusion between viral and cellular target membrane and is used as a crucial component of the broad-spectrum vaccine [6–8]. There are many strategies in the research of broad-spectrum HA vaccines, such as changing the ways of immunization and transforming HA proteins [3, 4, 9, 10]. The discovery of several broadly reactive antibodies against the HA head and stalk domain was a major milestone towards the development of universal influenza virus vaccine [11–13]. Previous studies showed that HAs from H3N2 have two conversed cysteines, in the 540 and 544 sites of the transmembrane domain, which were significant for the stability of HA trimer [14, 15]. The stable structure of HA trimer depended on disulfide bonds formed between cysteines of the HA monomer and was important for HA’s immunogenicity [14]. The introduction of a Cys-Phe-Leu-Leu-Cys (CFLLC) minidomain into the transmembrane domain of H1, H5 and H9 HA proteins by replacing with H3 HA transmembrane increased higher antibodies in mice than wild type [16–22]. Accordingly, the protein family of protein disulfide isomerases (PDIs) which assist the formation of disulfide bonds [23] were investigated for the ability to improve the stability of HA proteins in this study.

PDIs are a kind of enzyme that catalyzes redox reaction in the endoplasmic reticulum (ER), which can help the formation of disulfide bonds between cysteines and the correct folding of proteins [24–26]. There are more than 20 members of this protein family in mammals [23, 27]. Cells cannot survive if the important members are knocked out, like PDI-one of members [28]. In this study, we chose two members of the PDIs family: PDI and ERp57. PDI is one of the highest content proteins in the ER [25]. ERp57, unlike most PDI family members, does not contain a C-terminal ER retention motif capable of effectively limiting localization to the ER lumen and has been found in many different subcellular locations [29]. Available evidences indicated multiple distinct biological functions of ERp57, such as mediation of protein folding and Ca^2+^ balance in ER, delivery of apoptotic signaling in the mitochondria, and potential regulation of gene expression in the nucleus [29]. ERp57 was proved that it has a crucial role in post-translational reshuffling to the native set of HA disulfides [30].

However, it is unclear whether PDIs can actively improve the stability of the HA structure and whether the mice stimulated by HA with more stable HA trimers could generate the higher level of antibodies. In this study, we overexpressed two members of PDIs family: PDI and ERp57 to study their effect on the stability of HA, which results were further confirmed by the knockdown expression. Moreover, we investigated whether HA proteins from ERp57-overexpressed 293 T cells could stimulate the mice to produce higher antibodies than those from the conventional cells. Our results showed that ERp57 could improve the stability and immunogenicity of H3N2 HA, which may help the development of vaccine against influenza against pandemics.

## Materials and methods

### Cell lines, viruses, and mice

Human embryonic kidney cells (293 T) kept by our laboratory were cultured in Dulbecco’s modified Eagle’s medium supplemented with 10% fetal bovine serum (Thermo Scientific), penicillin (100 units/ml), and streptomycin (100 μg/ml) at 37 °C with 5% CO_2_.

Influenza virus strains A/swine/Guangdong/01/1998(H3N2), A/chicken/Guangdong/32/2007(H9N2) and A/Puerto Rico/8/1934(H1N1) were kept by our laboratory.

BALB/c mice were bred at the Laboratory Animal Center of Sun Yat-Sen University under the pathogen-free conditions. Six-eight-week-old female mice were randomly assigned to treatment or control groups in the experiment.

### Plasmid construction

RNA of H3N2 viruses (GenBank accession no. FJ830855.1) and 293 T cells were extracted using HiPure Viral RNA/DNA Kit (Magen) and TRIzol (Invitrogen) respectively. cDNA was prepared using PrimeScript™ II 1st Strand cDNA Synthesis Kit (TAKARA). H3 HA was amplified from H3N2 viruses using the following primers: H3 HA-Forward (*Kpn* I) 5′-GGGGTACCTAATTCTATCAACCATGAAGACTAT-3′ and H3 HA-Reverse (*Xho* I) 5′-CCGCTCGAGAGGGTGTTTTTAATTACTAATATACTCA-3′. PDI and ERp57 segments were amplified from 293 T cells using the following primers: PDI-Forward (*Hind* III) 5′-CCCAAGCTTCCAGGATTTATAAAGGCGAGGC-3′, PDI-Reverse (*Not* I) 5′-CGGAATTCCGGGTCTGGCTTTGCGTAT-3′, ERp57-Forward (*Hin*d III) 5′-CCCAAGCTTCGCAAGCAGCGGGTTAGT-3′ and ERp57-Reverse (*Bam*H I) 5′-CGGGATCCTCCTAGTCCTCCCCAATGGTT-3′. H3 HA, PDI and ERp57 PCR products were cloned into pcDNA3.1(+) (Invitrogen) respectively. All clones were verified by sequencing (Thermo).

### Expression of HA proteins with PDIs overexpression in 293 T cells

To investigate whether PDIs can improve the stability of HA, cells were transfected with 2 μg of H3 HA and PDI (or ERp57) plasmids in a 6-well plate, using PEI-MAX (Polyscience) according to the manufacturer’s protocol. Briefly, 293 T cells were seeded in a 6-well plate at a density of 1 × 10^5^ cells/mL and cultured until the cells reached approximately 80–90% confluence. Purified plasmids and PEI-MAX (Polyscience) were incubated in the opti-MEM at the concentration ratio of 1:4 respectively at room temperature for 5 min and then mixed to incubate at room temperature for 20 min. The mixture was added to 293 T cells to incubate at 37 °C for 8 h with 5% CO_2_ before the replacement with the fresh medium. After 48 h incubation at 37 °C with 5% CO_2_, 293 T cells were collected.

### Expression of HA proteins with ERp57 RNA depletion by small interfering RNA (siRNA)

To downregulate ERp57 expression, 293 T cells were transfected with 200 nM siRNA against ERp57 (5′-GGACAAGACTGTGGCATAT-3′, RIBOBIO) using lipo2000 (Invitrogen) according to the manufacturer’s instructions. Briefly, 293 T cells were seeded in a 12-well plate at a density of 1 × 10^5^ cells/mL and cultured until the cells reached approximately 50% confluence. siRNA and lipo2000 at the concentration ratio of 1:2 were incubated in the opti-MEM respectively at room temperature for 5 min and then mixed to incubate at room temperature for 20 min. After the mixture was added to 293 T cells to incubate at 37 °C for 24 h with 5% CO_2_, 293 T cells were transfected with 1 μg H3 HA plasmids in a 12-well plate using PEI-MAX. After incubation for 48 h at 37 °C with 5% CO_2_, 293 T cells were collected.

### Characterization of H3 HA and PDIs expression

To confirm and characterize H3 HA and PDIs expression, real-time qPCR, western-blot, immunofluorescence assay, and flow cytometry were carried out.

For real-time qPCR, total RNA was extracted from 293 T cells with TRIzol, and treated with DNase I. cDNA was synthesized by reverse transcription using RT-PCR kit (TaKaRa, Dalian). Real-time qPCR was performed with the following primers (ERp57-Forward 5′- TCACGGACGACAACTTCGAG-3′, ERp57-Reverse 5′- GTTGGCAGTGCAATCAACCT-3′, GAPDH-Forward 5′- AACGGATTTGGTCGTATTG-3′, GAPDH-Reverse 5′- GGAAGATGGTGATGGGATT-3′) using LightCycler 480 SYBR Green I Master (Roche) according to the manufacturer’s protocol. Briefly, PCR was performed in a 20 μL volume containing 100 ng of cDNA, 10 μL of 2 × LightCycler 480 SYBR Green I Master, and a 0.2 μM of each gene-specific primer. The thermal cycling parameters were referring to the manufacturer’s protocol: 95 °C for 5 min, 40–45 cycles of 95 °C for 10 s, 58 °C for 20 s, 72 °C for 30 s, 1 cycles of 95 °C for 5 s, 65 °C for 1 min, 97 °C for continuous, and 40 °C for 10 s. The final cycle was set to obtain a melting curve for the PCR products to determine the specificity of the amplification. The GAPDH gene was utilized as the reference gene. Expression levels of genes were calculated relative to the expression of the GAPDH gene and expressed as fold increase or decrease relative to the control samples.

For western-blot, cells were collected and lysed with RIPA lysis buffer. The protein concentration in samples was quantified by BCA assay (Thermo Fisher Scientific). And then samples were heated at 100 °C for 10 min in the loading buffer with (for reducing SDS-PAGE) or without (for non-reducing SDS-PAGE) 1% β-mercaptoethanol. Total proteins were separated by 10% SDS polyacrylamide gels, electrophoretically transferred to polyvinylidene difluoride membrane (Millipore, 45 μm), and then detected with mouse polyclonal anti-H3 HA serum (kept by our lab), rabbit polyclonal anti-PDI antibody (Abcam, ab3672), mouse monoclonal anti-ERp57 antibody (Abcam, ab13506) respectively, followed with the corresponding secondary antibody and the commercial ECL kit (Fdbio science). Protein loads were estimated using murine anti-β-actin mAb (Abmart, M20010). The band intensity was quantified using ImageJ software.

For immunofluorescence assay, after 48 h transfection, cells were collected and fixed in 4% phosphate-buffered paraformaldehyde at room temperature for 10–15 min, permeabilized, blocked, and reacted with primary antibodies (same as those used in western-blot) and mouse-specific Cy3-conjugated secondary antibody/rabbit-specific FITC-conjugated secondary antibody (PTG). Cell nuclei were stained with 4′-6′-diamidino-2-phenylindole (DAPI) (Sigma-Aldrich) and visually inspected by epifluorescence light microscopy before the acquisition of representative areas by confocal microscopy. Stained sections were analyzed by a scanning confocal microscopy (Leica TCS-SP5).

For flow cytometry, cells were collected in a single-cell suspension with the 0.05% Trypsin-EDTA digestion, fixed in 70% ethanol in the ice for 30 min, permeabilized, blocked, and reacted with primary antibodies (same as those used in western-blot) and secondary antibodies (same as those used in immunofluorescence assay). Flow cytometric analyses were performed using FACS Calibur (BD biosciences), and data were analyzed using FlowJo software.

### Purification of H3 HA proteins

H3 HA proteins were purified by using ion-exchange chromatography as described previously [31]. Briefly, samples were lysed in Buffer A (20 mM sodium phosphate, 1.0 mM EDTA, 0.01% Tergitol-NP9, 5% glycerol, pH 5.89) in the ice for 30 min and centrifugated at 10,000×g for 25 min. The supernatant was loaded on tandem Q/SP columns and the columns were disconnected after washed by buffer A. HA was eluted from the SP column with buffer B (20 mM sodium phosphate, 0.03% Tergitol-NP9, 5% glycerol, pH 7.02) and buffer C (20 mM sodium phosphate, 150 mM NaCl, 0.03% Tergitol-NP9, 5% glycerol, pH 7.02) consecutively, but mostly in the buffer C. The eluted HA proteins in buffer C was further purified and concentrated by ultrafiltration with a stirred cell using a 100 kDa MWCO regenerated cellulose membrane (Millipore).

### Mouse immunization and challenge

Six- to eight-week-old female BALB/c mice (*n* = 5) were vaccinated subcutaneously twice at the interval of 2 weeks with 10 μg of H3 HA expressed in ERp57-overexpressed 293 T cells (named as H3 HA/ERp57) or in the conventional 293 T cells (named as H3 HA) in a 100 μl volume with oil emulsion containing 2% aluminum stearate, 2% Tween-80 and 4% span 80. Blood samples were collected from the tail vein of each mouse 2 weeks after the second immunization. After one more week, mice were challenged intranasally with 40 μL of mouse-adapted wild-type H1N1 viruses at a dose of 3 × 50% mice lethal dose (MLD_50_). After the challenge, the mice were monitored for 14 days for survival and weight loss daily. The mice that lost 25% or more of their initial body weight were scored dead and euthanized.

### Hemagglutination inhibition (HI) assay

HI assay was performed as described before [14]. Briefly, four HA units of inactivated H1N1, H3N2, and H9N2 viruses were used. Each serum sample was treated with receptor destroying enzyme (RDE, Denka Seiken) at 37 °C overnight, then incubated at 56 °C for 1 h immediately for the hydrolysis of RDE. Serum samples were diluted 10-fold initially then 2-fold continuously for ten times. The highest dilution of serum samples that inhibits hemagglutination was defined as the HI titers.

### Enzyme-linked immunosorbent assay (ELISA) for testing HA-specific IgG antibodies

HA specific IgG antibody titers were tested by ELISA as previously described [32]. Briefly, inactivated H1N1, H3N2, and H9N2 purified viruses at the concentration of 1 μg/mL of HA1 were coated at 4 °C overnight, incubated with serial dilutions of each serum sample at 37 °C for 1 h, and detected by HRP-conjugated goat anti-mouse IgG polyclonal antibodies (Proteintech). The optical densities were detected at 450 nm (OD_450_) using ELISA plate reader (Bio-Tek Instruments). The antibody titer was determined as the reciprocal of the highest dilution of three-fold OD_450_ reading of negative control.

### Statistics analysis

All statistical analyses were performed using Graphpad Prism software (Graphpad). Statistical analyses were performed using one or two-way ANOVA. Data are shown as mean ± SD or mean ± SEM as indicated in the figure legends. Significance was assumed with *p < 0.05, **p < 0.01, ***p < 0.001, ****p < 0.0001.

## Results

### Overexpression of PDIs did not impact the expression and characterization of HA proteins in 293 T cells

**Fig. 1:**
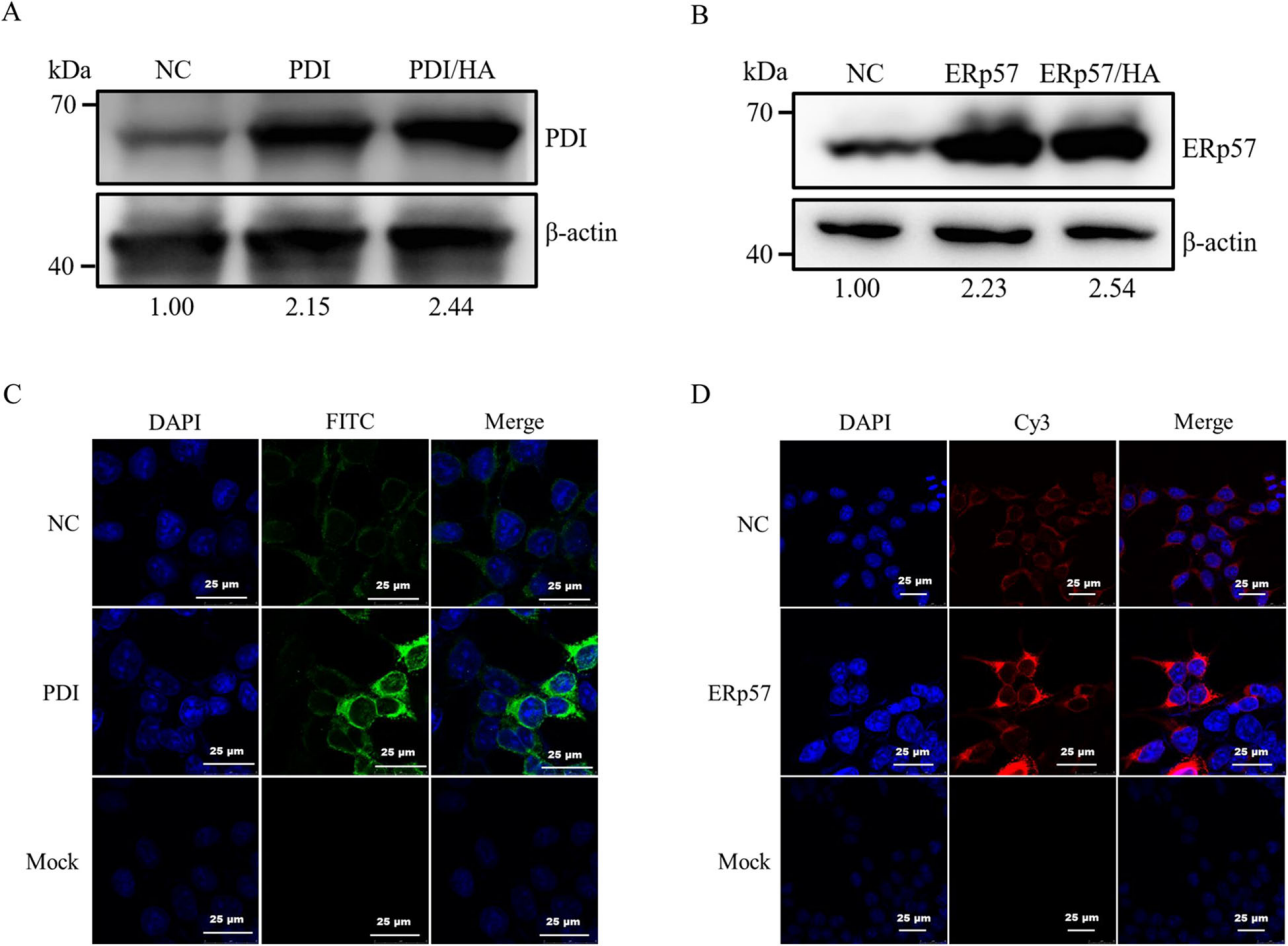
Overexpression and characterization of PDI proteins in 293 T cells. NC denotes the normal culture of 293 T cells; PDI and ERp57 denote overexpression of PDI and ERp57 proteins respectively; PDI/HA and ERp57/HA denote co-expression of H3 HA with ERp57 and PDI proteins respectively. **a** Western-blot analysis of PDIprotein expression under reducing condition. **b** Western-blot analysis of ERp57 protein expression under reducing condition. ImageJ was used to quantify PDIs. **c** Indirect immunofluorescence assay of PDI protein expression. **d** Indirect immunofluorescence assay of ERp57 protein expression. Cells nuclei were stained with DAPI (blue). PDI/ERp57 proteins were reacted with primary antibody and FITC/Cy3-conjugated secondary antibody (green/red). Scale bars indicate 25 μm

**Fig. 2:**
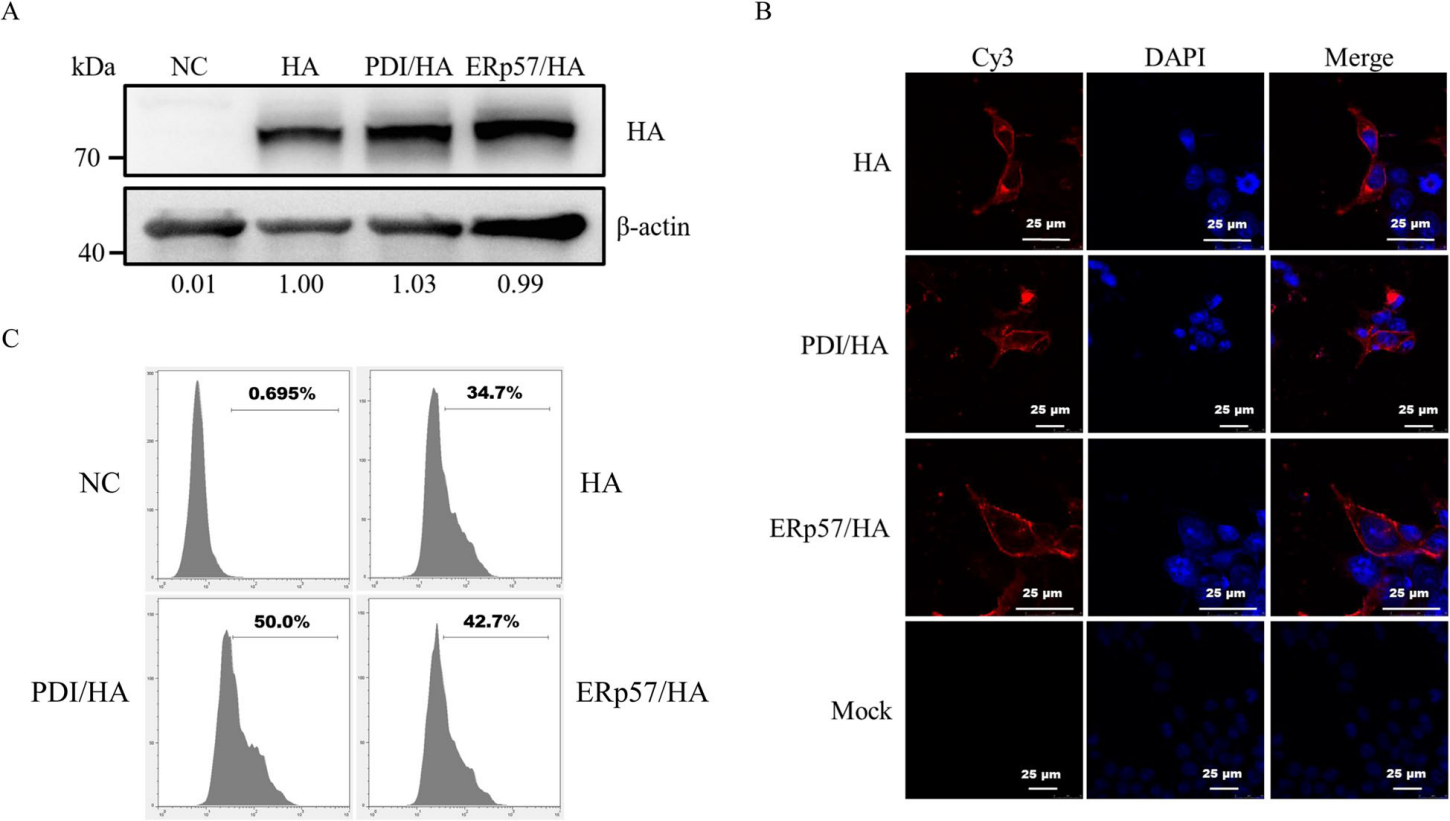
Expression and characterization of HA proteins in 293 T cells. HA denotes the single expression of H3 HA proteins; PDI/HA denotes co-expression of PDI and H3 HA proteins; ERp57/HA denotes co-expression of ERp57 and H3 HA proteins. **a** Western-blot analysis of HA protein expression under reducing condition. ImageJ was used to test the quantity of HA proteins. **b** Indirect immunofluorescence assay of HA protein expression. Cells nuclei were stained with DAPI (blue), and HA proteins were reacted with primary antibody and Cy3-conjugated secondary antibody (red). Scale bars indicate 25 μm. **c** Flow cytometry analysis of HA protein expression

To investigate the impact of the overexpression of PDIs on the expression of H3 HA proteins, the recombinant H3 HA and PDIs plasmids were co-transfected into 293 T cells. PDI and ERp57 proteins were successfully overexpressed in 293 T cells which was confirmed by western-blot (Fig. 1a and b) and immunofluorescence assay (Fig. 1c and d). The percentage of cells showing overexpression was 54.9% for PDI and 48.9% for ERp57 (Fig. 1c and d). PDI and ERp57 were abundant in overexpression groups but sparse in negative-control group. The expression of H3 HA proteins was not affected by the overexpression of PDI and ERp57 in 293 T cells as demonstrated by western-blot (Fig. 2a), immunofluorescence assay (Fig. 2b) and flow cytometry (Fig. 2c).

### HA trimers showed significantly higher stability in PDIs-overexpressed cells than in the conventional 293 T cells

**Fig. 3:**
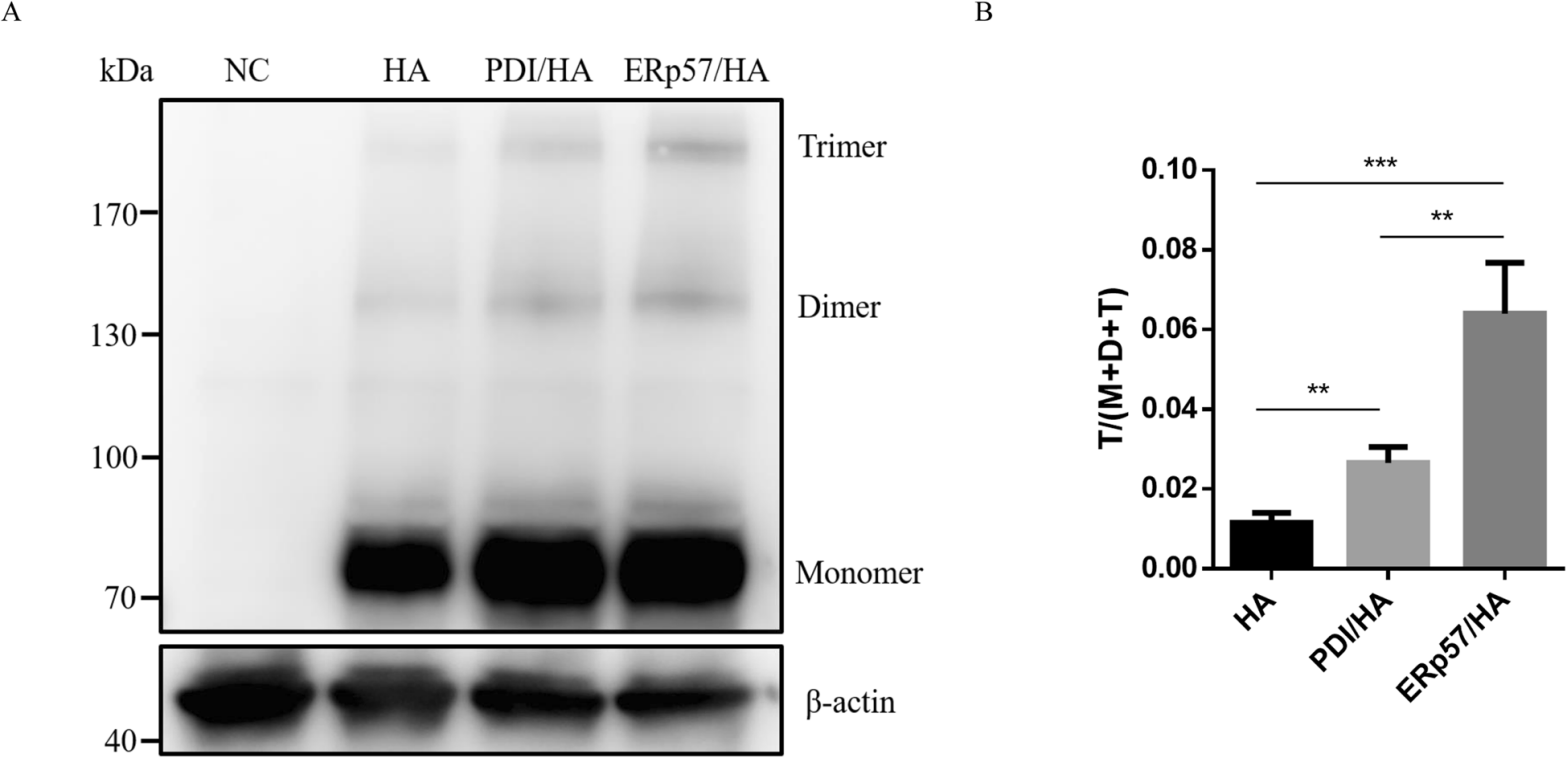
The percentage of the disulfide-bonded HA trimers significantly increased in the PDI-overexpressed 293 T cells. **a** Western-blot analysis of the disulfide-bonded HA trimers under non-reducing condition. **b** A statistical graph of the disulfide-bonded HA trimers analyzed by ImageJ. T denotes Trimer, D denotes Dimer, and M denotes Monomer. ** represents *P* < 0.01, *** represents *P* < 0.001

To investigate the stability of HA, the disulfide-bonded HA trimers were detected by western-blot under non-reducing condition (Fig. 3a). The results showed that the proportion of the disulfide-bonded HA trimers in PDIs-overexpressed 293 T cells (11.1% ± 2.8 for PDI/HA group, 22.4% ± 5.4 for ERp57/HA group) was significantly higher than those in the conventional 293 T cells (4.0% ± 2.3) (*p* < 0.05, *p* < 0.01)(Fig. 3b), which means that HA trimers expressed in the PDIs-overexpressed 293 T cells showed the higher stability than those expressed in the conventional 293 T cells. What’s more, the percentage of HA trimers expressed in the ERp57-overexpressed 293 T cells was significantly higher than those in the PDI-overexpressed cells (*p* < 0.05), suggesting ERp57 is more effective to stabilize HA proteins than PDI. Therefore, we selected ERp57 for the following research.

### The percentage of HA trimers significantly descended when the ERp57 expression was knocked down by siRNA

**Fig. 4:**
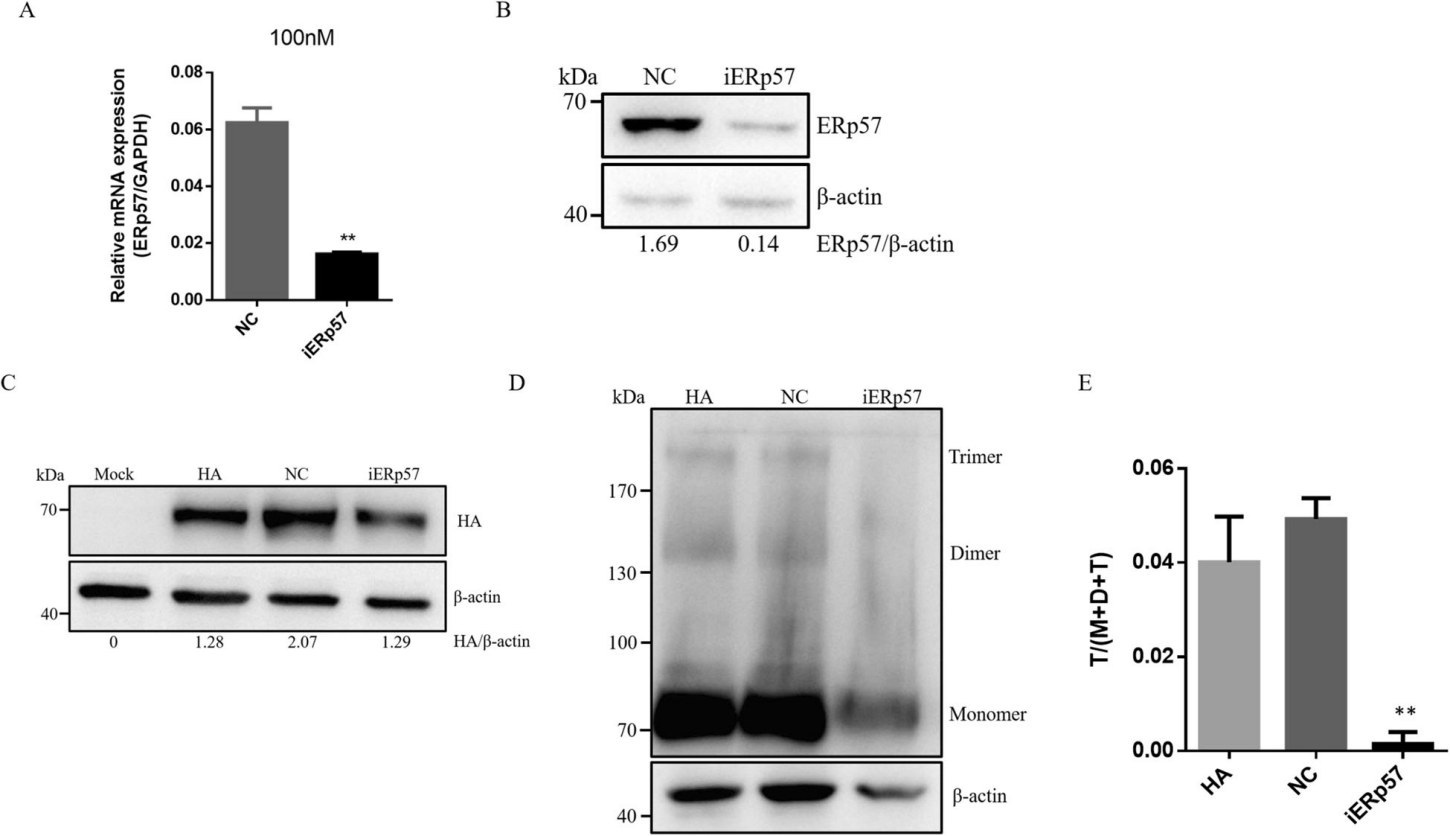
The percentage of the disulfide-bonded HA trimers significantly descended when the ERp57 proteins were down-expressed. **a** Real-time qPCR analysis of ERp57 gene expression. iERp57 denotes the expression of ERp57 genes in the ERp57 gene-knockdowned cells. ** represents *P* < 0.01. **b** Western-blot analysis of ERp57 expression under reducing condition; iERp57 denotes the expression of ERp57 proteins in the ERp57 gene-knockdowned cells. **c** Western-blot analysis of HA expression under reducing condition; Mock denotes the conventional 293 T cells transfected by blank plasmid; HA denotes the expression of HA proteins in the conventional 293 T cells; NC denotes the expression of HA proteins in the 293 T cells transfected by negative control siRNA which don’t interference any genes expression in the 293 T cells; iERp57 denotes the expression of HA proteins in the ERp57 gene-knockdowned cells. **d** Western-blot analysis of the disulfide-bonded HA trimers under non-reducing condition; iERp57 denotes the expression of HA proteins in the ERp57 gene-knockdowned cells. **e** A statistical graph of the disulfide-bonded HA trimers analyzed by ImageJ. *** represents *P* < 0.001

To investigate the stability of HA expressed in the ERp57-downexpressed and conventional 293 T cells, we down-regulated the expression of ERp57 by siRNA. Synthesized siRNA targeting ERp57 efficiently decreased the expression of endogenous ERp57 mRNA (Fig. 4a) and protein (Fig. 4b). The results of western-blot under reducing condition showed that the amount of HA proteins was not significantly different between groups (Fig. 4c). The disulfide-bonded HA trimers were detected by western-blot under non-reducing condition (Fig. 4d), and the results showed the proportion of the disulfide-bonded HA trimers was significantly lower (0.1% ± 0.3, *p* < 0.01) (Fig. 4e) when ERp57 proteins expressed in a low level, suggesting that ERp57 proteins are important for maintaining the stability of HA.

### H3 HA from ERp57-overexpressed 293 T cells elicited significantly higher HI titers and HA-specific antibodies than H3 HA from the conventional cells

To explore whether or not HA proteins from ERp57-overexpressed 293 T cells show higher immunogenicity, mice were immunized subcutaneously twice with the vaccine H3 HA/ERp57 and H3 HA that contained HA proteins from the ERp57-overexpressed and conventional 293 T cells, respectively. The serum samples were analyzed by HI assay and ELISA.

**Fig. 5:**
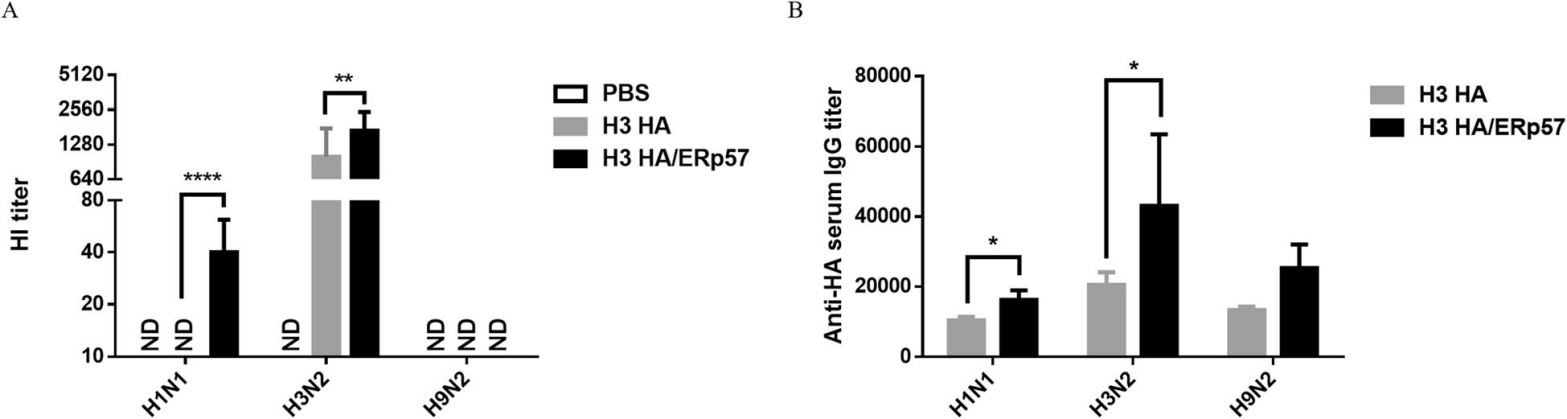
H3 HA/ERp57 elicited significantly higher HA-specific antibodies than H3 HA. H1N1, H3N2, and H9N2 influenza viruses were purified and inactivated as antigens for assay. BALB/c mice (*n* = 5) were immunized twice with H3 HA or H3 HA/ERp57 or PBS as control. **a** The HI titer against homologous (H3N2) and heterologous (H1N1 or H9N2) antigens. The collected serum samples were assayed for HI titers following the conventional HI assay. ND denotes HI antibody not detectable. **b** The HA-specific antibodies against homologous (H3N2) and heterologous (H1N1 or H9N2) antigens. * represents *p* < 0.05, ** represents *p* < 0.01, **** represents *p* < 0.0001

H3 HA/ERp57 elicited 1.68-fold higher HI titers than H3 HA against H3N2 antigen (1004.2 ± 769.5 vs 1688.4 ± 700.3, *p =* 0.0047). H3 HA/ERp57 elicited HI titers against H1N1 (40 ± 21.9), while H3 HA did not elicit HI titer against H1N1 (*p* < 0.0001). HI titer against H9N2 in H3 HA/ERp57 and H3 HA group were not detected (Fig. 5a).

No matter for the homologous and heterologous antigen, H3 HA/ERp57 elicited higher serum HA-specific IgG antibody titers than H3 HA. The HA-specific IgG antibody titers of H3 HA and H3 HA/ERp57 did not show statistical difference for the heterologous antigen H9N2 (13,324.2 ± 929.1 vs 25,333.3 ± 6694.4, *p* = 0.1636), while H3 HA/ERp57 elicited significantly higher HA-specific IgG antibody titers than H3 HA for the heterologous antigen H1N1 (16,266.0 ± 2604.4 vs 10,435 ± 910.5, *p* = 0.0221) and homologous antigen H3N2 (46,436.7 ± 24,770.1 vs 20,517.5 ± 3563.7, *p* = 0.0204) (Fig. 5b).

### Mice immunized with H3 HA/ERp57 showed better resistance and higher survival rate against heterologous H1N1 challenge than those immunized with H3 HA

**Fig. 6:**
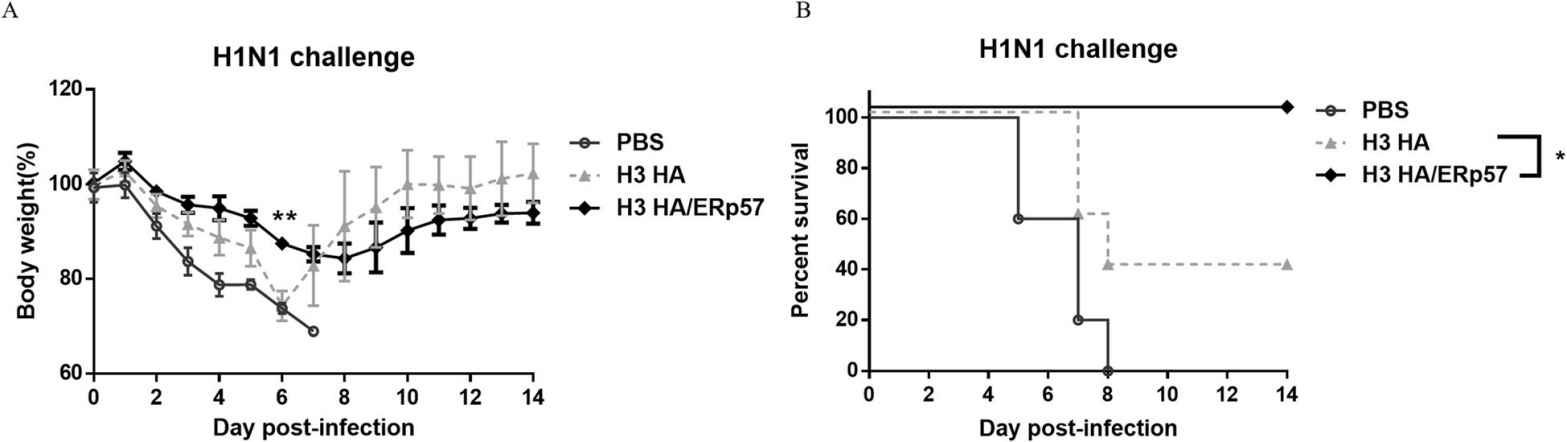
Mice immunized with H3 HA/ERp57 showed less body weight loss against heterologous (H1N1) challenges than those immunized with H3 HA. BALB/c mice (n = 5) immunized with H3 HA or H3 HA/ERp57 were infected i.n. by 3 × MLD_50_ H1N1 viruses 3 weeks after the second immunization. The mice were monitored daily for their survival rates and body weight for 14 days. The body weight percentage was calculated according to the formula of (retained body weight/initial body weight) × 100%. The survival rate was calculated according to the formula of (number of survival mice/number of challenged mice) × 100%. **a** Weight loss of immunized mice against infection of H1N1 influenza virus, ** represents *p* < 0.01. **b** Survival rates of immunized mice against infection of H1N1 influenza virus, * represents *p* < 0.05

To determine the level of protection against the challenge of the heterologous virus, mice were challenged intranasally with 3 × MLD_50_ of mouse-adapted wild-type H1N1 viruses 2 weeks after final immunization and monitored for their weight loss and survival daily for 14 days. Mice immunized with H3 HA/ERp57 showed less weight loss than those immunized with H3 HA till 7 day post-infection (dpi), especially at 6 dpi (*p* = 0.0035) (Fig. 6a), indicating that mice immunized with H3 HA/ERp57 showed the better resistance against H1N1 viruses than those immunized with H3 HA. And mice receiving H3 HA/ERp57 were completely protected against H1N1 challenge while those receiving H3 HA had only 40% survival rate (*p* = 0.0486) (Fig. 6b).

## Discussion

The broad-spectrum vaccine with wider protection against influenza viruses is a hot topic in recent years. HA, as the main ingredient of broad-spectrum vaccine, plays an important role on the improvement for immunogenicity of vaccines [1–3, 33–35]. Many researches showed that the stable structure of HA was crucial for HA’s immunogenicity [14, 15, 36] However, how to improve HA stability effectively and safely still needs more researches. Here, we uncovered a key role of PDIs in the improvement of the stability of HA*.* HAs from overexpressed-PDIs 293 T cells were more stable than those from the conventional cells and could stimulate mice to produce higher HA-specific IgG and HI antibodies against H1N1 and H3N2 viruses.

The research of HA protein, which is related to the immunogenicity and infectivity of the influenza virus, is mainly focused on its structure, function, and application in the vaccine [13, 37, 38]. In order to improve influenza vaccines, many efforts have been made to stabilize the expressed HA proteins, such as fusing HA extracellular domain to the trimerization sequences or introducing a CFLLC minidomain into the transmembrane domain of HA proteins [16, 18, 20, 34, 39–41]. PDIs, one of the most important protein families in cells, catalyze the formation of intra-and inter-molecular disulfide bonds between cysteines and help the correct folding of proteins [24–26]. ERp57 has been proved to play a crucial role in the post-transcription of HA proteins [30]. In this study, we demonstrated the stability of HAs from the overexpressed-PDIs 293 T cells increased significantly. The expression level of PDI proteins was low in 293 T and CEF cells than in Sf9 and MDCK cells (Fig. S1). CEF cells as the primary cells are difficult to obtain while 293 T cells are often used to express heterologous proteins, so we overexpressed PDIs in 293 T cells to study the effect of PDIs on HA proteins. Overexpression of PDIs did not impact the expression and characterization of HA proteins in 293 T cells as demonstrated by western-blot, immunofluorescence assay, and flow cytometry. HA proteins from PDIs-overexpressed 293 T cells showed a higher proportion of the disulfide-bonded HA trimers than those from the conventional cells, and knockdown of ERp57 by siRNA significantly decreased the percentage of HA trimers, which demonstrated that PDIs can promote the formation efficiency of disulfide bonds of HA proteins and improve the stability of HA in 293 T cells. Furthermore, the proportion of the disulfide-bonded HA trimers from ERp57-overexpressed 293 T cells was significantly higher than PDI-overexpressed cells. The results showed that increased PDI expression favors the stability of HA, suggesting that correct protein folding may be crucial to the stability of the expressed HA proteins in 293 T cells.

Many studies have shown the close correlation of HA stability and immunity. By fusing HA extracellular domain to the trimerization sequences, the stability and immunogenicity of HA proteins have been increased [34, 39–41]. By introducing a CFLLC minidomain into the transmembrane domain of H1, H5, H7 and H9 HA proteins, these HA proteins expressed in insect cells increased their stability, cross-reactive immunity and protection over their wildtype counterparts [16, 18, 20]. In this study, HA proteins from ERp57-overexpressed 293 T cells stimulated the mice to generate the higher HA-specific IgG against H1N1 and H3N2 viruses than those from the conventional cells. These results demonstrated that HA proteins from ERp57-overexpressed 293 T cells had better immunogenicity than those from the conventional cells. As shown in Fig. 5a, H3 HA/ERp57 elicited 1.68-fold higher HI titers than H3 HA against H3N2 antigen (*p* = 0.0047). H3 HA/ERp57 elicited HI titers against H1N1 while H3 HA did not elicit HI titer against H1N1 (*p* < 0.0001). So, H1N1 was chosen as the challenged strain. Mice immunized with H3 HA/ERp57 showed less body weight loss than those immunized with H3 HA till 7 dpi, especially at 6 dpi (*p* < 0.01), and mice in H3 HA/ERp57 groups were completely protected against H1N1 challenge, while mice in H3 HA groups were only 40% protected. So further studies are necessary to confirm the exact contribution of PDIs to the stability of HA in other influenza viruses or in other frequently-used HA-expressed systems and understand the exact underlying mechanism.

In summary, this study demonstrated PDIs can improve the formation efficiency of the disulfide bonds between HA monomers and increase the stability of HA. Furthermore, HAs from ERp57-overexpressed 293 T cells could stimulate higher HI and IgG antibodies against H1N1 and H3N2 antigens. And mice receiving HAs from ERp57-overexpressed 293 T cells showed the better resistance against H1N1 viruses and higher survival rate than mice receiving HAs from conventional 293 T cells.

## Conclusion

Conclusively, ERp57, a member of the PDIs family, could improve the stability and immunogenicity of H3 HA, which may help the development of vaccine against influenza against pandemics.

## Supplementary information

**Additional file 1: Figure S1.** The expression of PDI in different cell lines.

## Data Availability

Data and materials are available from the corresponding author on reasonable request.

## Abbreviations

HA: Hemagglutinin
PDI: Protein disulfide isomerases
ER: Endoplasmic reticulum
CFLLC: Cys-Phe-Leu-Leu-Cys
IFA: Immunofluorescence assay
MLD_50_: 50% mice lethal dose
HI: Hemagglutination inhibition
ELISA: Enzyme-linked immunosorbent assay
dpi: Day post-infection.
