# Supplementary figures and images for: Disulfide isomerase ERp57 improves the stability and immunogenicity of H3N2 influenza virus hemagglutinin
Source: Virol J. 2020 Apr 21;17:55. doi: 10.1186/s12985-020-01325-x (PMC7175539; doi:10.1186/s12985-020-01325-x)

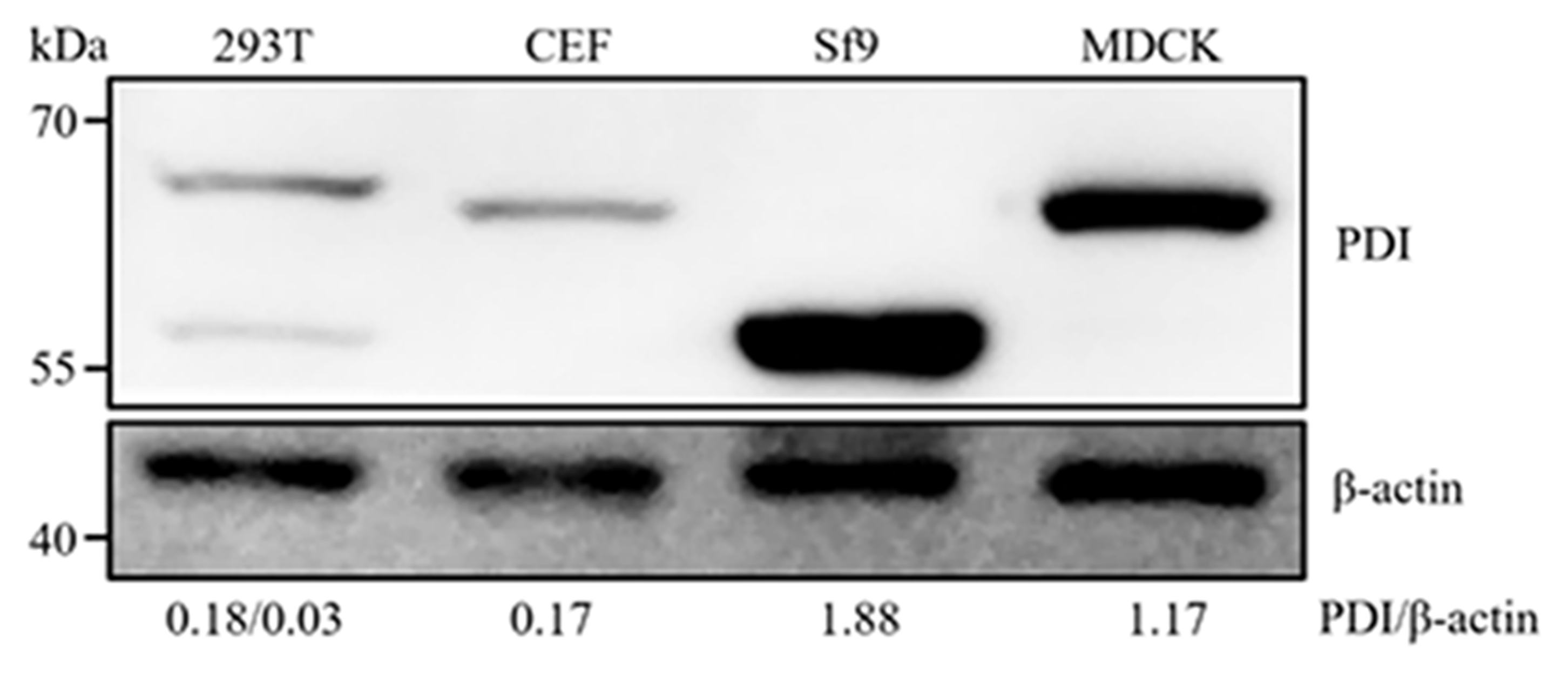

Supplement: Supplementary file 1 — Additional file 1: Figure S1. The expression of PDI in different cell lines. [file 12985_2020_1325_MOESM1_ESM.tif]
